# Exploratory Analysis of iPSCS-Derived Neuronal Cells as Predictors of Diagnosis and Treatment of Alzheimer Disease

**DOI:** 10.3390/brainsci10030166

**Published:** 2020-03-13

**Authors:** Eugenio Cavalli, Giuseppe Battaglia, Maria Sofia Basile, Valeria Bruno, Maria Cristina Petralia, Salvo Danilo Lombardo, Manuela Pennisi, Reni Kalfin, Lyubka Tancheva, Paolo Fagone, Ferdinando Nicoletti, Katia Mangano

**Affiliations:** 1 Department of Biomedical and Biotechnological Sciences, University of Catania, Via S. Sofia 89, 95123 Catania, Italy; eugeniocavalli9@hotmail.it (E.C.); sofiabasile@hotmail.it (M.S.B.); salvo.lombardo.sdl@gmail.com (S.D.L.); manuela.pennisi@unict.it (M.P.); ferdinic@unict.it (F.N.); kmangano@unict.it (K.M.); 2 University Sapienza, Piazzale A. Moro, 5, 00185 Roma, Italy; giuseppe.battaglia@uniroma1.it (G.B.); valeria.bruno@uniroma1.it (V.B.); 3 IRCCS Neuromed, Località Camerelle, 86077 Pozzilli (IS), Italy; 4 Department of Educational Sciences, University of Catania, 95100 Catania, Italy; m.cristinapetralia@gmail.com; 5 Institute of Neurobiology, Bulgarian Academy of Sciences, Acad. G. Bonchev Str., Block 23, 1113 Sofia, Bulgaria; reni_kalfin@abv.bg (R.K.); lyubkatancheva@gmail.com (L.T.)

**Keywords:** Alzheimer disease, Induced pluripotent stem cells-derived neuronal cells, drug repurposing, biomarkers

## Abstract

Alzheimer’s disease (AD) represents the most common neurodegenerative disorder, with 47 million affected people worldwide. Current treatment strategies are aimed at reducing the symptoms and do slow down the progression of the disease, but inevitably fail in the long-term. Induced pluripotent stem cells (iPSCs)-derived neuronal cells from AD patients have proven to be a reliable model for AD pathogenesis. Here, we have conducted an in silico analysis aimed at identifying pathogenic gene-expression profiles and novel drug candidates. The GSE117589 microarray dataset was used for the identification of Differentially Expressed Genes (DEGs) between iPSC-derived neuronal progenitor (NP) cells and neurons from AD patients and healthy donors. The Discriminant Analysis Module (DAM) algorithm was used for the identification of biomarkers of disease. Drugs with anti-signature gene perturbation profiles were identified using the L1000FWD software. DAM analysis was used to identify a list of potential biomarkers among the DEGs, able to discriminate AD patients from healthy people. Finally, anti-signature perturbation analysis identified potential anti-AD drugs. This study set the basis for the investigation of potential novel pharmacological strategies for AD. Furthermore, a subset of genes for the early diagnosis of AD is proposed.

## 1. Introduction

Alzheimer’s disease (AD) represents the most common neurodegenerative disorder, with 47 million affected people worldwide. AD is characterized by several neuropathological changes—including cerebral atrophy, intense synaptic loss, and neuronal death—in regions of the prefrontal cortex and hippocampus that are responsible for cognitive functions. The disease shows a prodromal period that can last for decades and is characterized by a preclinical asymptomatic phase before cognitive impairment occurs [1]. Mild cognitive impairment (MCI) represents the first clinical phase of AD, characterized by an alteration in episodic memory. It is reasonable to believe that treatment with disease-modifying agents would likely be most effective in this stage of AD, before neurodegeneration is too marked and widespread. Thus, studies aimed at identifying potential AD biomarkers for early diagnosis is warranted. However, MCI can derive from a variety of causes (e.g., vascular, presence of Lewy bodies) and only approximately half of cases is associated to AD, with consequent obvious diagnostic difficulties. Indeed, magnetic resonance imaging (MRI)-based regional brain volumes, cerebrospinal fluid (CSF) analytes, and positron emission tomography (PET) imaging of cerebral fibrillar β-amyloid along ad hoc cognitive tests have been investigated as biomarkers of disease, they are useful only for the late stages of the disease, and may not be sensitive enough to detect initial neuropathophysiological processes occurring in AD patients who show mild cognitive impairment [2,3]. Hence for most cases, definite diagnosis is only possible with the post-mortem analysis of the brain and with the observation of severe brain atrophy and neuronal loss, as well as the presence of dense extracellular deposits and intracellular aggregates within neurons, identified as amyloid plaques and neurofibrillary tangles, respectively.

As regards AD therapy, up to now, only five drugs have been approved by FDA for human use. However, none of them are able to cure the disease and are only modestly able to slow down AD progression and improve the cognitive abilities of the patients. The reason for the lack of an effective treatment for AD likely relies on the multifactorial pathology of this disease, as well as the heterogeneous patient population [4]. Therefore, there is a strong need to develop novel anti-AD therapies. However, traditional drug development is burdened by the requirement of long time, high financial investments, and low success rate. In recent years, a large number of in vitro and in vivo studies, as well as some clinical studies, have been carried out with the aim of evaluating protective effects of some known multitarget molecules with antioxidant, anti-inflammatory, and neuroprotective potential on neurodegenerative processes [5]. On the other hand, drug repurposing can be used to redirect approved drugs for treating different disorders and seems an attractive strategy in AD, as it may expedite the design of phase II-III clinical trials, reduce the risks associated with early stages of drug development, while being cost-effective. Fessel et al. proposed that the combination of eight drugs that are already approved for different clinical indications and with limited or null overlapping activities that may warrant preclinical studies in animal models or Phase II PoC studies in humans [6]. Clearly, daily combination of eight drugs is clinically difficult and a trial of this kind would require adequate compliance of patients and also considerable economic supports that may be difficult to obtain in view of the lack of adequate patent protection of these drugs in the area of AD [6].

With the aim to identify possible diagnostic and therapeutic (e.g., theranostic) markers of AD development and progression, we have presently used a machine learning approach to identify a subset of genes that may predict AD in Induced Pluripotent Stem Cells (iPSC)-derived neuronal cells from dermal fibroblasts. The generation of iPSCs derived neuronal cells from patients with AD represent a unique opportunity to create a relevant in vitro model for mechanistic studies and preclinical drug discovery, and have been widely exploited in AD [7,8,9,10,11,12], as well as in other diseases such as amyotrophic lateral sclerosis [13], Parkinson’s disease [14], Rett syndrome [15], schizophrenia [16], Duchenne muscular dystrophy, Becker muscular dystrophy, Down syndrome, Juvenile diabetes mellitus, Huntington disease and Lesch-Nyhan syndrome [17].

**Figure 1:**
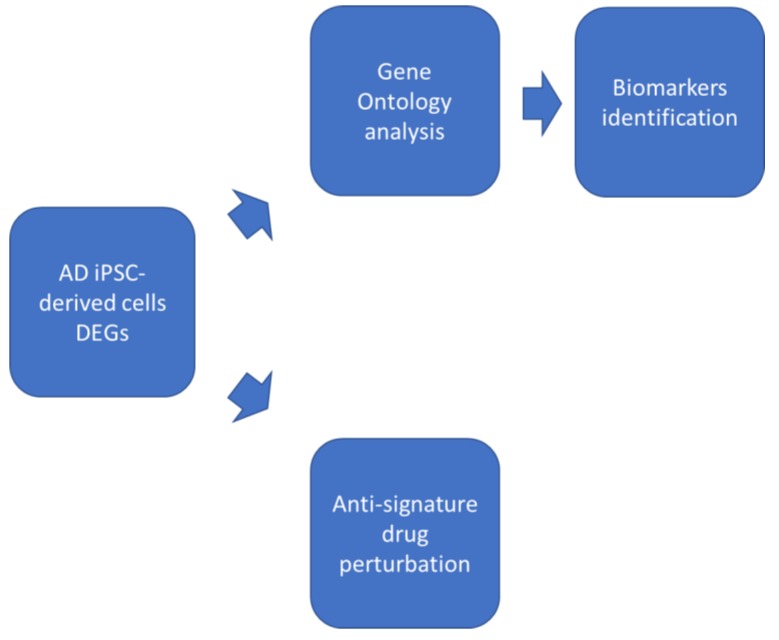
Study plan.

Furthermore, we have performed a computational analysis of candidate drugs, based on their ability to modulate in an opposite manner the transcriptional profiles characterizing AD, in order to shortlist promising anti-AD drugs. A diagram showing the study plan is presented as Figure 1.

## 2. Material and Methods

### 2.1. Dataset Selection

The publicly available microarray dataset GSE117589, originally generated and analyzed by Meyer and collaborators [1] was used for the identification of Differentially Expressed Genes (DEGs) between Induced Pluripotent Stem Cells (iPSC)-derived Neural Progenitor cells (NPCs) and neurons from AD patients and healthy donors. GSE117589 was retrieved from the Gene Expression Omnibus (GEO) databank (https://www.ncbi.nlm.nih.gov/gds) [1]. Briefly, for the generation of the dataset, iPSCs were obtained by retroviral transduction of KLF4, SOX2, c-MYC, and OCT4 in human dermal fibroblasts (Coriell Cell Repository, Camden, NJ, USA) from 5 healthy donors and 5 individuals with sporadic AD (SAD). Cells were then differentiated into NPCs and neurons, as described in the Meyer et al., 2019 [1]. The age of the healthy donors was 72.2 ± 13.3 and the age of the SAD patients was 69.6 ± 11.1. The female to male ratio was 2/3 and 3/2 in the healthy controls and SAD patients, respectively. All healthy donors had the E3/E3 *APOE* genotype, with the exception for one subject, who had the E3/E4 genotype. Two SAD patients had the E3/E3 genotype, two had the E4/E4 genotype and one the E2/E3 genotype [1]. Transcriptomic profiling was performed using the Affymetrix U133 Plus 2.0 arrays. The submitter-supplied pre-preprocessed and normalized gene expression matrix was used for the analysis [1]. Briefly, the probesets from the U133 Plus 2.0 platform were first converted into Ensembl genes and gene ids without annotation were removed [1]. Raw data were then preprocessed using the Robust Multi-array Average (RMA) algorithm [1].

### 2.2. Identification of Biomarkers of Disease and Validation

For the identification of the Differentially Expressed Genes (DEGs) in the cells from SAD individuals and Healthy donors, the LIMMA (Linear models for microarray data) parametric test was used. An adjusted *p*-value < 0.1 was considered to indicate a statistically significant difference. Gene Ontology (GO) analysis was performed for the DEGs, using the Metascape web-based tool, using default settings [18].

In order to identify a specific transcriptomic signature able to discriminate healthy subjects from AD patients, we used the Discriminant Analysis Module (DAM) algorithm [19]. DEGs were used as input data. DAM performs first a gene dimensional reduction method, the Multivariate Partial Least Squares (MPLS). Afterwards, the Polychotomous Discriminant Analysis (PDA) was applied as classification method. Hierarchical Clustering (HCL) was performed using the identified predictors in order to determine the relative distance of samples using Pearson’s correlation as similarity comparison.

In order to validate the results from the biomarkers prediction, we interrogated the GSE118553 microarray dataset [20]. This dataset was chosen as it included whole-genome expression data of brain areas known to be affected by AD pathology (i.e., entorhinal cortex, temporal cortex, and frontal cortex) and an area partially spared by the disease (i.e., cerebellum) from healthy controls (*n* = 27) and AD patients (*n* = 52) [20]. Not all subjects had tissue samples extracted from all four brain regions [20]. Entorhinal cortex AD patients were 83.9 ± 9.7 years old (vs. 71.9 ± 15.6 of control subjects), had a Braak stage of 4.9 ± 1 and a disease duration of 11.8 ± 5.2 years. Temporal cortex AD patients were 82.7 ± 9.8 years old (vs. 71.5 ± 16.9 of controls subjects), had a Braak stage of 4.9 ± 0.9 and a disease duration of 9.7 ± 5.4 years. Frontal cortex AD patients were 82.5 ± 4.7 years old (vs. 69.8 ± 15.4 of controls subjects), had a Braak stage of 4.9 ± 1 and a disease duration of 10.5 ± 5.7 years. Cerebellum AD patients were 82.6 ± 10.6 years old (vs. 69.4 ± 16 of controls subjects), had a Braak stage of 5.1 ± 0.3 and a disease duration of 9.4 ± 5.6 years. Principal Component Analysis (PCA) was used to evaluate the segregation of the samples using the predicted biomarkers.

### 2.3. Drug Prediction Analysis

The L1000FDW web-based utility [21] was used to identify potential novel pharmacological strategies for the treatment of AD. L1000FWD calculates the similarity between an input gene expression signature vector and the LINCS-L1000 data, in order to rank drugs potentially able to reverse the transcriptional signature [21]. The L1000 transcriptomic database is part of the Library of Integrated Network-based Cellular Signatures (LINCS) project, a NIH Common Fund program, that extended the Connectivity Map project and includes the transcriptional profiles of approximately 50 human cell lines upon exposure to about 20,000 compounds, over a range of concentrations and time [21]. An adjusted *p*-value (*q*-value) of 0.05 has been considered as threshold for statistical significance.

### 2.4. Statistical Analysis

GraphPad Prism (v. 8) and MeV (v. 4.9) software programs were used for the statistical analysis and the generation of the graphs. Differentially expression analysis, PCA and DAM have been performed using the MeV 4.9 software, which used R v.2.11.1 and LIMMA v3.4.5.

## 3. Results

### 3.1. Machine Learning-Identified Genes for the Diagnosis of AD

**Figure 2:**
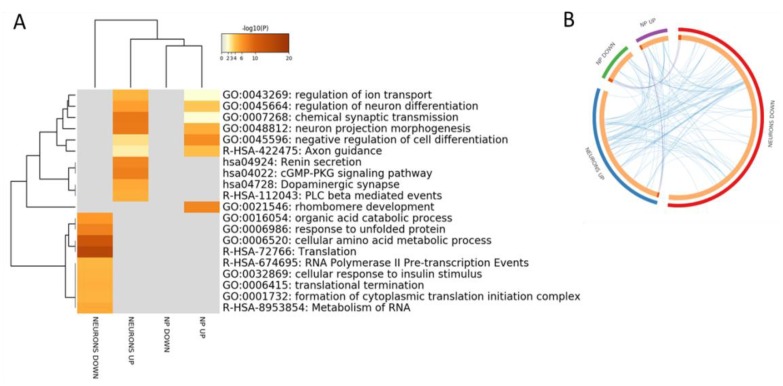
(**A**) Hierarchical clustering of the top 20 most enriched terms by genes significantly modulated in Induced pluripotent stem cells (iPSCs)-derived neuronal progenitors cells (NP) and iPSCs-derived neurons from sporadic Alzheimer’s disease patients vs. healthy donors. The heatmap is colored by the p-values, and grey cells indicate the lack of significant enrichment; (**B**) Circos plot showing overlapping between the genes significantly modulated in iPSCs-derived neuronal progenitors cells (NP) and iPSCs-derived neurons from sporadic Alzheimer’s disease patients vs. healthy donors. Purple lines link the same genes that are shared by the input lists. Blue lines link the different genes that fall in the same ontology term.

In order to identify a specific gene signature characterizing AD, we first interrogated the GSE117589 microarray dataset. LIMMA analysis identified 65 DEGs in NP cells from SAD patients as compared to Healthy controls, 30 upregulated and 35 downregulated. When analyzing iPSC-derived neurons, 386 DEGs were found, 131 upregulated and 255 downregulated in SAD patients as compared to Healthy controls. Gene Ontology analysis revealed a partial overlapping of enriched biological processes among the upregulated DEGs in AD NP cells and neurons, that included “regulation of ion transport”, “regulation of neuron differentiation”, “chemical synaptic transmission”, “neuron projection morphogenesis”, “negative regulation of cell differentiation” and “axon guidance” (Figure 2A,B).

Among the DEGs identified for the iPSC-derived neurons, five have been associated to AD by GWAS: SPON1, ANKRD55, RHOBTB22, TTLL7 and MRPL10. With the exception of MRPL10, which is downregulated, all of the other genes are upregulated in AD samples. None of the DEGs identified in iPSC-derived NP cells have been associated to AD.

**Figure 3:**
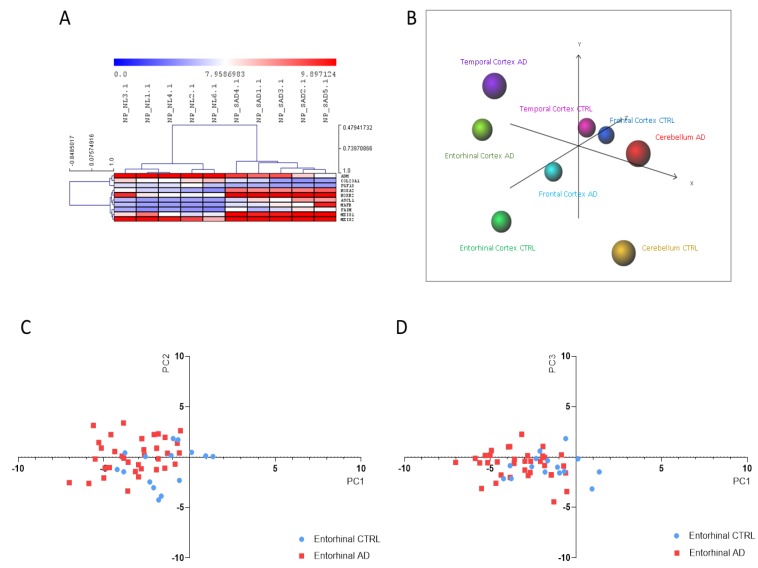
(**A**) Hierarchical clustering of the Alzheimer’s disease (AD) biomarkers identified using the Discriminant Analysis Module (DAM) algorithm in the Induced pluripotent stem cells (iPSCs)-derived neuronal progenitors (NP) cells from sporadic Alzheimer’s disease patients vs. healthy donors (CTRL); (**B**) Principal Component Analysis (PCA) using the identified AD biomarkers on the samples from the GSE118553 dataset; (**C**) Scatterplot showing Principal Component (PC)1 and PC2 for the entorhinal samples from the GSE118553 dataset; (**D**) Scatterplot showing PC1 and PC3 for the entorhinal samples from the GSE118553 dataset.

Next, we employed the DAM analysis, in order to identify the lowest number of genes able to differentiate SAD patients from healthy individuals. A total of 10 predictors out of the 65 NP cells DEGs were identified from the DAM analysis. Consistent with these findings, HCL accurately segregated iPSC-derived NP cells from SAD patients from those obtained from non-demented controls (Figure 3A). The 10 identified predictors are presented in Table 1. In order to validate the reliability of the identified biomarkers, we performed a PCA on the entorhinal, frontal and temporal cortex, as well as on cerebellum, from healthy controls and AD patients. As shown in Figure 3B–D, a discrete separation of samples from healthy and AD subjects was observed for the entorhinal cortex. Only a partial segregation was observed for the temporal and frontal cortex (Figure 3B). An overlapping distribution of samples was instead observed for the cerebellum (Figure 3B).

**Figure 4:**
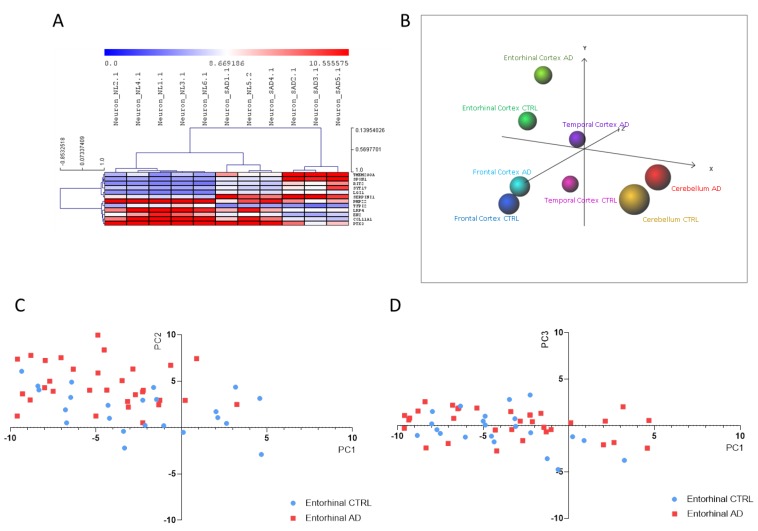
(**A**) Hierarchical clustering of the Alzheimer’s disease (AD) biomarkers identified using the Discriminant Analysis Module (DAM) algorithm in the Induced pluripotent stem cells (iPSCs)-derived neurons from sporadic Alzheimer’s disease patients (SAD) vs. healthy donors; (**B**) Principal Component Analysis (PCA) using the identified AD biomarkers on the samples from the GSE118553 dataset; (**C**) Scatterplot showing PC1 and PC2 for the entorhinal samples from the GSE118553 dataset; (**D**) Scatterplot showing PC1 and PC3 for the entorhinal samples from the GSE118553 dataset.

As regards iPSC-derived neurons, DAM analysis identified 12 predictors out of 386 DEGs. Consistent with these findings, HCL accurately segregated iPSC-derived neurons from SAD patients from those obtained from non-demented controls (Figure 4A). The 12 identified predictors are presented in Table 2 and were used to perform a PCA analysis on samples of entorhinal, frontal and temporal cortex, as well as of cerebellum, from healthy controls and AD patients, obtained from the GSE118553 dataset. As shown in Figure 4B–D, a discrete separation of samples from healthy and AD subjects was observed.

### 3.2. Prediction of Novel Chemotherapeutics for AD

**Figure 5:**
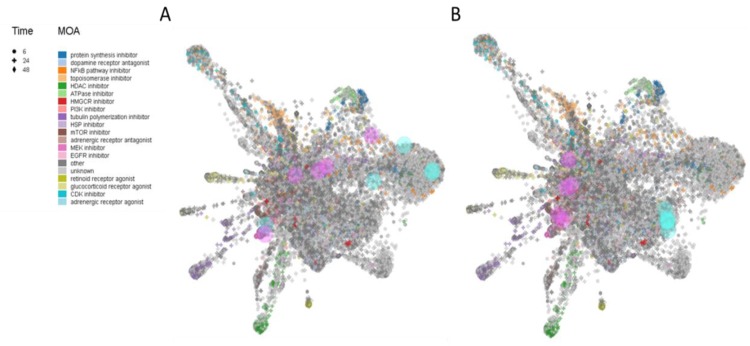
L1000FDW visualization of drug-induced signature. Input genes are represented by the significantly upregulated and downregulated genes obtained from the analysis of the GSE117589 dataset, for iPSC-derived NP cells (**A**) and neuronal cells (**B**). Blue and red circles identify drugs with similar and anti-similar signatures. Dots are color-coded based on the Mode of Action (MOA) of the respective drug.

Anti-signature perturbation analysis was performed using the DEGs identified for the iPSCs-derived NP cells and neurons (Figure 5A,B, respectively). Among the significant predicted drugs, we have prioritized those already in clinical use. In Table 3, we have enlisted the potential anti-AD drugs identified by the L1000FWD analysis using the iPSC-derived NP cells model of AD. Among them, the top three drugs are: etacrynic-acid, a diuretic; cytarabine, a chemotherapy medication used to treat acute myeloid leukemia, acute lymphocytic leukemia, chronic myelogenous leukemia and non-Hodgkin’s lymphoma; and betamethasone, a corticosteroid. Table 4 contains a list of the potential anti-AD drugs identified using the iPSC-derived neuronal cells model of AD. Among them, the top three drugs are: cyclosporin-a, an immunesuppressive agent; dabrafenib, a B-raf inhibition used to treat melanoma; and penfluridol, indicated for antipsychotic treatment of schizophrenia and psychotic disorders. Interestingly, from our analysis, cyclosporin-a is the only drug that has been convergently predicted using both iPSC-derived NP and neuronal cells (Table 3 and Table 4).

## 4. Discussion

Given the limited access to brain-derived neuronal cells, little information is still available on the pathogenic processes that characterize the initial phases of sporadic AD. Therefore, the use of in-vitro-based models that reflect AD-affected neurons may allow for early diagnosis, and to test preventive approaches for patient treatment. Recently, independent groups have differentiated cells from AD patients into neuronal progenitors and neuronal cells using iPSC-based methods, and evaluated them for the molecular mechanisms underlying disease development. These studies are thought to give valuable insights regarding AD molecular phenotypes, and could represent predictive models to be used in the future in a clinical setting.

We have here identified a gene-signature that could be used for the diagnosis of AD, by using a publicly available whole-genome transcriptomic dataset on iPSC-derived NP cells and neurons from AD patients and non-demented controls. Our analysis followed a more conservative approach than those used by Meyer and collaborators [1], resulting in a lower number of prioritized DEGs.

Interestingly, we observed that the identified AD biomarkers allowed to differentially segregate brain samples from healthy subjects and AD patients. When using the biomarkers identified using the NP cells, a better segregation was observed for the entorhinal cortex, while a poor segregation was observed for frontal cortex, temporal cortex and cerebellum. This is in line with the observation from Patel and collaborators [20] who described a higher percentage of perturbed genes in the entorhinal cortex, followed by progressively reduced numbers of DEGs in the temporal cortex, frontal cortex and, finally in the cerebellum [20]. This seems to reflect the pattern of AD progression and suggests that the iPSC-based model used in the present analysis may better mirror ab initio transcriptional defects underlying AD pathogenesis. Furthermore, gene ontology analysis revealed that these genes are involved in the regulation of cell differentiation and neurogenesis. These data support the hypothesis that the early identification of susceptible individuals is possible using iPSCs-based models. Furthermore, the biomarkers predicted using the iPSC-derived neurons showed a similar ability to discriminate AD from non-demented patients.

It is believed that early interventions that tackle factors that are associated and increase the relative risk of AD development, could drastically reduce the burden of dementia associated with AD at the population level. Indeed, such interventions could reduce the development of signs and symptoms of AD, preventing the progression from MCI to AD, and reducing the subclinical deficits in dementia individuals.

Typically, development of a new drug takes up to 15 years and requires between 2 to 3 billion dollars of investment. In addition, on average, only 10% of drugs entering phase I trials obtain approval for human use. The rest of the molecules are dropped because of toxicity issues or lack of efficacy [22,23]. Drug repurposing, i.e., finding novel indications for already approved drugs, overcomes these limitations, as toxicity, pharmacokinetic and pharmacodynamic are already available and consequently, the drugs can rapidly be tested in phase II-III trials, dramatically reducing development risk, time and cost. Nowadays, almost 30% of new drugs are repositioned drugs [22]. Drug repurposing can be investigated both experimentally and computationally (in silico) [24]. The latter is based on the evaluation of the anti-similarity between drugs and a disease [25,26,27]. To this aim, gene expression signatures obtained from -omics data [28] are used to discover novel mechanisms of disease and searches inverse drug–disease relationships by matching gene expression profiles. We and others have used whole-genome expression databases for the better understanding of pathogenic pathways and the prediction of diagnostic and therapeutic strategies for a series of disorders—e.g., immunoinflammatory and autoimmune diseases [29,30,31,32,33,34,35,36,37], and cancer [38,39]—which has led to the identification of potential novel therapeutic targets [40,41,42,43,44,45,46,47,48,49,50,51]. However, gene perturbation alone cannot accurately predict treatment options due to variability related to disease genetics and epigenetics, as well as, experimental settings. For instance, although arginase inhibitor was expected to increase neurotoxicity, in preclinical model of AD, it has been shown to exert protection in mice [52]. Limitations of our work rely on the fact that our model does not account for epigenetic and post-transcription modifications affecting the final phenotype. Furthermore, although drug gene perturbation signatures come from genes ubiquitously modulated across a series of cell lines, however, they are constructed on cell types strikingly different from those found in the central nervous system, and treatments are limited in terms of concentrations and time points. Finally, drug candidates for AD, and neurodegenerative disease in general, should also be selected on the basis on their ability to cross the blood–brain barrier.

Interestingly, in our study, cyclosporine-a was predicted to be a potential anti-AD drug, when using both the iPSC-derived NP cells and neurons. This is in line with recent data from Stallings’ group, showing that cyclosporine-a blocked dendritic spine loss in Aβ42-treated cells [53]. Furthermore, cyclosporine-a inhibited amyloid synthesis and improved amyloid induced neurotoxicity in neuroblastoma cells [54]. Finally, a pilot open-label study of tacrolimus, which shares the same mode of action with cyclosporin-a in AD (ClinicalTrials.gov Identifier: NCT04263519) is expected to be completed by December 2021.

In addition, our analysis has identified the corticosteroids, betamethasone and triamcinolone, as potential anti-AD drugs. This seems consistent with a post mortem study conducted by Beeri et al. [55] on 694 brains of subjects who did not have neuropathologies other than neuritic plaques (NPs), neurofibrillary tangles (NFTs), or cerebrovascular disease, that patients receiving corticosteroids had significantly lower ratings and counts of NPs for all neuropathological measures, and NFTs overall and in the cerebral cortex and amygdala. In contrast, no significance was observed for subjects who received NSAIDs. AD has been linked to neuroinflammation [56], and biochemical and neuropathological studies on AD brains provide evidence for the activation of inflammatory pathways and glial inflammation [57]. Notably, women are more susceptible to developing immunoinflammatory disorders than men [58], and accordingly, the estimated lifetime risk of developing AD shows a female to male ratio of 1.8. Based on these observations, the nasal administration of corticosteroids has been proposed for the early stages of AD [59]. On the other hand, preclinical studies have shown conflicting effects of glucocorticoids on CNS, as hypersecretion was shown to contribute to age-related hippocampal degeneration [60].

## 5. Conclusions

Our study set the basis for the identification of biomarkers for the early diagnosis of AD, using the low invasive model of iPSC-derived neuronal cells. Indeed, the use of imaging techniques or the measurement of CSF markers is difficult to achieve for the costs and invasive procedures. Furthermore, blood biomarkers have not yet given satisfactory results as diagnostic tools in AD. However, this is an exploratory study and future studies on larger cohorts of patients with SAD are needed to validate the data here generated. Furthermore, since other cellular types—including astrocytes and microglia—are likely to be directly involved in the etiopathogenesis of AD, future studies aimed at investigating potential glial-related biomarkers are warranted. Finally, the single and combined administration of the potential anti-AD drugs that has emerged from our study seems worthy being evaluated in preclinical models of AD to exploit the translatability of these findings to the clinical setting.

## Figures and Tables

**Table 1:** List of biomarkers identified by DAM analysis in iPSC-derived NP cells ^1^.

Gene Stable ID	Gene Name	Gene Description
ENSG00000134138	MEIS2	Meis homeobox 2
ENSG00000105996	HOXA2	homeobox A2
ENSG00000050767	COL23A1	collagen type XXIII alpha 1 chain
ENSG00000156427	FGF18	fibroblast growth factor 18
ENSG00000173917	HOXB2	homeobox B2
ENSG00000139352	ASCL1	achaete-scute family bHLH transcription factor 1
ENSG00000148926	ADM	adrenomedullin
ENSG00000204103	MAFB	MAF bZIP transcription factor B
ENSG00000143995	MEIS1	Meis homeobox 1
ENSG00000158234	FAIM	Fas apoptotic inhibitory molecule

^1^DAM: Discriminant Analysis Module; iPSC: induced Pluripotent Stem Cell; NP: neuronal progenitor.

**Table 2:** List of biomarkers identified by DAM analysis in iPSC-derived neuronal cells ^1^.

Gene Stable ID	Gene Name	Gene Description
ENSG00000060718	COL11A1	collagen type XI alpha 1 chain
ENSG00000103528	SYT17	synaptotagmin 17
ENSG00000105825	TFPI2	tissue factor pathway inhibitor 2
ENSG00000108231	LGI1	leucine rich glioma inactivated 1
ENSG00000109099	PMP22	peripheral myelin protein 22
ENSG00000134569	LRP4	LDL receptor related protein 4
ENSG00000152214	RIT2	Ras like without CAAX 2
ENSG00000163536	SERPINI1	serpin family I member 1
ENSG00000163661	PTX3	pentraxin 3
ENSG00000164484	TMEM200A	transmembrane protein 200A
ENSG00000164778	EN2	engrailed homeobox 2
ENSG00000262655	SPON1	spondin 1

^1^DAM: Discriminant Analysis Module; iPSC: induced Pluripotent Stem Cell; NP: neuronal progenitor.

**Table 3:** Potential anti-AD drugs identified by the L1000FWD analysis using the iPSC-derived NP cells model of AD.

Drug	Similarity Score	*p*-Value	*q*-Value	Z-Score	Combined Score	Category
etacrynic-acid	−0.1739	1.36E-06	5.49E-03	1.8	−10.58	sodium/potassium/chloride transporter inhibitor
cytarabine	−0.1522	6.38E-06	1.29E-02	1.74	−9.02	ribonucleotide reductase inhibitor
betamethasone	−0.1522	1.69E-05	1.29E-02	1.85	−8.81	glucocorticoid receptor agonist
triamcinolone	−0.1522	2.32E-05	1.38E-02	1.84	−8.52	glucocorticoid receptor agonist
flecainide	−0.1304	2.14E-04	2.73E-02	1.65	−6.05	sodium channel blocker
econazole	−0.1304	1.57E-04	2.65E-02	1.85	−7.03	lanosterol demethylase inhibitor, sterol demethylase inhibitor
cyclosporin-a	−0.1304	1.59E-04	2.65E-02	1.84	−6.99	calcineurin inhibitor

**Table 4:** Potential anti-AD drugs identified by the L1000FWD analysis using the iPSC-derived neuronal cells model of AD.

Drug	Similarity Score	*p*-Value	*Q*-Value	Z-Score	Combined Score	Category
cyclosporin-a	−0.0954	2.84E-10	6.41E-07	1.64	−15.7	calcineurin inhibitor
dabrafenib	−0.0954	1.82E-11	1.12E-07	1.84	−19.78	RAF inhibitor
penfluridol	−0.0954	3.91E-11	1.53E-07	1.83	−19.02	T-type calcium channel blocker
niclosamide	−0.0916	5.26E-10	1.13E-06	1.82	−16.9	DNA replication inhibitor, STAT inhibitor
lasalocid	−0.0878	2.56E-09	3.42E-06	1.77	−15.22	bacterial permeability inducer
triclosan	−0.084	1.82E-08	1.43E-05	1.79	−13.84	antibacterial agent
progesterone	−0.084	6.17E-10	1.26E-06	1.66	−15.31	progesterone receptor agonist
artesunate	−0.0802	1.87E-08	1.43E-05	1.66	−12.81	DNA synthesis inhibitor
selamectin	−0.0802	2.32E-08	1.63E-05	1.7	−12.95	nematocide
